# Mass distribution of azithromycin and child mortality among underweight infants in rural Niger: a subgroup analysis of the AVENIR cluster-randomised trial

**DOI:** 10.1136/bmjopen-2024-097916

**Published:** 2025-03-27

**Authors:** Brittany Peterson, Ahmed Arzika, Ramatou Maliki, Amza Abdou, Bawa Aichatou, Ismael M Bello, Diallo Beidi, Nasser Galo, Nasser Harouna, Alio M Karamba, Sani Mahamadou, Moustapha Abarchi, Almou Ibrahim, Elodie Lebas, Zijun Liu, Carolyn Brandt, Emily Colby, Catherine E Oldenburg, Travis C Porco, Benjamin Arnold, Thomas M Lietman, Kieran Sunanda O’Brien

**Affiliations:** 1 F. I. Proctor Foundation, University of California, San Francisco, California, USA; 2 Department of Epidemiology and Biostatistics, University of California, San Francisco, California, USA; 3 Centre de Recherche et Interventions en Santé Publique, Birni N’Gaoure, Niger; 4 Programme Nationale de Santé Oculaire, Niamey, Niger; 5 Department of Ophthalmology, University of California, San Francisco, California, USA; 6 Institute for Global Health Sciences, University of California, San Francisco, California, USA

**Keywords:** Mass Drug Administration, Mortality, Community child health

## Abstract

**ABSTRACT:**

**Objective:**

Azithromycin has been shown to reduce all-cause child mortality. This subgroup analysis investigates azithromycin’s mortality impact by underweight status using *Azithromycine pour la Vie des Enfants au Niger: Implementation et Recherche* (AVENIR) trial data.

**Design:**

The AVENIR trial randomised communities into three arms: azithromycin for children aged 1–59 months, azithromycin for infants aged 1–11 months or placebo. Weight-for-age z-score was used to categorise children into subgroups of either moderate to severe underweight or not and severe underweight or not.

**Setting:**

2880 communities with a population of less than 2500 people in the Dosso and Tahoua regions of Niger that participated in the AVENIR trial were included.

**Participants:**

97 572 children aged 1–59 months who had weight captured during at least one census participated.

**Results:**

Underweight subgroups had higher overall mortality compared with non-underweight subgroups. IRDs of deaths in children aged 1–11 months comparing communities receiving azithromycin to children 1–59 months of age to placebo were −6.2 deaths per 1000 person-years (95% CI −9.3 to −2.6) overall, −8.0 (95% CI −15.9 to −0.4) in the moderate to severe subgroup and −11.2 (95% CI −26.0 to −2.1) in the severe subgroup. Similar trends were noted in the azithromycin 1–11 month comparison. Malnutrition was not a statistically significant effect modifier for either comparison.

**Conclusions:**

Although analyses suggest the potential for stronger effects in more severe underweight subgroups, we were unable to demonstrate underweight status as an effect modifier. In fact, azithromycin mass drug administration to children 1–59 months old reduced mortality in all subgroups, and, especially as the number of lives saved would be the highest by treating all subgroups, our results do not support restricting eligibility for this intervention.

**Trial registration number:**

clinicaltrials.gov NCT04224987.

**STRENGTHS AND LIMITATIONS OF THIS STUDY**

- The trial included systematic data collection of weight for all children aged 1–11 months.
- The cluster-randomised design of the *Azithromycine pour la Vie des Enfants au Niger: Implementation et Recherche* trial precluded bias in estimation of mortality effects.
- The inclusion of two regions in Niger led to greater generalisability of the results.
- The scale of the study limited the ability to include other indicators of malnutrition such as wasting and stunting.
- The study was underpowered to detect differences among subgroups given the focus on 1–11 month mortality, which represents a small subgroup and a rare outcome.

## Introduction

 Several cluster-randomised trials in sub-Saharan Africa have shown that mass drug administration (MDA) of biannual azithromycin to children 1–59 months old leads to lower under-five mortality rates compared with administering placebo.^1^,^3^ West and Central Africa exhibit a persistently high burden of under-five mortality.^4^,^7^ Azithromycin MDA for child survival thus presents a promising approach to addressing this. However, the intervention increases the prevalence of antimicrobial resistance (AMR) in communities receiving azithromycin.^8^,^11^ Limiting the number of children who receive antibiotics with this intervention could decrease selection for AMR and might be possible through targeting children with the highest mortality risk.

Malnutrition plays a large part in child mortality in sub-Saharan Africa. The risk of mortality in malnourished children increases through the presence of infectious diseases such as diarrhoeal and respiratory disease.^12^ Further, children infected with bacterial pathogens can be at an increased risk of malnutrition through appetite reduction and nutrient deficiency.^13^ Antibiotics are routinely used as part of treatment programmes for children with severe acute malnutrition.^14^,^17^ Previously, the MORDOR study in Niger suggested that mortality reduction following MDA may be greater among children 1–11 months old classified as underweight using weight-for-age z-score (WAZ) thresholds of both −2 and −3, although the trial was not powered to detect interaction.^18^ While this result generated the hypothesis that underweight children may experience greater benefit of MDA, the trial still found a larger number of deaths averted when treating all children regardless of malnutrition status.

*Azithromycine pour la Vie des Enfants au Niger: Implementation et Recherche* (AVENIR) is a cluster-randomised controlled trial in Niger that compared all-cause mortality in communities randomised to azithromycin MDA to children 1–59 months old, azithromycin MDA to children 1–11 months old or placebo. The study design provides an additional opportunity to examine effect modification by underweight status on child mortality, as the trial included a systematic data collection on weight with a larger sample size than prior studies across two regions in Niger. This prespecified secondary analysis leverages data from the AVENIR trial to determine whether there is a differential effect of azithromycin MDA on infant mortality in subgroups defined by moderate or severe underweight at the time of treatment.

## Methods

### Trial design, setting and participants

AVENIR was a response-adaptive cluster-randomised controlled trial comparing the effects of azithromycin MDA on child mortality in communities randomised to three arms: (1) azithromycin to children 1–59 months old, (2) azithromycin to children 1–11 months old with placebo to children 12–59 months old and (3) placebo to all children aged 1–59 months. The study protocol and main trial results have been published.^3 19^ This analysis focuses on comparisons between the azithromycin 1–59 month arm versus placebo arm and the azithromycin 1–11 month arm versus placebo arm. Communities included in the trial were rural and peri-urban with a population of 250–2499 located in the Dosso and Tahoua regions of Niger. Children were eligible for treatment if they were 1–59 months of age, weighed at least 3 kg and had no known allergy to macrolide antibiotics. Ethical approval for the study was obtained from Comité National Éthique pour la Recherche en Santé in Niger and the Institutional Review Board at the University of California, San Francisco. Verbal consent was obtained from community leaders before trial activities began and from the heads of households and guardians of each child at each study visit. For children 30–42 days old, written consent was also obtained. The trial was registered at clinicaltrials.gov (NCT04224987) on 13 January 2020.

### Data collection

Data were collected via a biannual census that occurred at the household of each participating family. (dataset)^20^ Trained census workers travelled door-to-door collecting data electronically using a custom CommCare application (Dimagi, Cambridge, Massachusetts, USA). All households in the community were approached for participation. Census information included demographic data for heads of household, guardians of children in the household and children aged 1–59 months, including tracking vital status at each visit follow-up. Children eligible for treatment were treated at the time of census. Weight was captured for all children 1–11 months old who participated in treatment using a hanging scale (ADE M111600, GmbH & Co., Hamburg, Germany). The trained census worker collecting the data for that household weighed the child and entered the child’s weight in kilograms to the nearest 0.1 kg. Census workers were trained to ensure the child is only wearing light clothing, hang the scale on its hook, wait until the scale shows ‘0.00’ and then add the child to the weight holder and capture weight.

### Interventions

Treatment was administered as either azithromycin (Pfizer, New York, USA) or matching placebo as a single 20 mg/kg oral dose. Children aged 1–11 months had their dose determined by a hanging scale (ADE M111600, GmbH & Co., Hamburg, Germany), and children 12–59 months old had their dose determined by a height-based dosing pole.

### Randomisation and masking

Communities were randomised to one of the three treatment arms. The trial used a response-adaptive allocation at the third and fourth phase of the trial when enrolling new communities. This was done to increase the likelihood that new communities would receive the more effective treatment enhancing equity within the study while still maintaining statistical validity. More regarding this response-adaptive allocation is detailed in the primary results paper.^3^ The randomisation sequence was generated by one unmasked biostatistician on the team, and all other members of the team including data analysts, data collectors, investigators and trial participants were masked. Both the treatment and placebo were packaged identically to aid masking.

### Outcomes

The outcome for this study was the infant mortality incidence rate as measured by the count of deaths from all causes per community and the person-time at risk in children 1–11 months old. A child was classified as a death if they were marked as alive in the beginning of one census interval and died the next. Person-time at risk was measured as the time spent alive from the beginning of one census interval to the end. Children marked as died contributed half the amount of time from that interval, and children marked as moved or unknown contributed no time.

### Assessment of nutritional status

Nutritional status was determined at the beginning of each census interval. All children aged 1–11 months had their weight calculated at the time of treatment to determine dosing. Weight was recorded in the mobile application to the nearest 0.1 kg. WAZ was calculated using 2006 WHO guidelines through the statistical package ‘anthro’ in R (R Foundation for Statistical Computing, Vienna, Austria).^21^,^23^ WAZ was used to define two subgroups: moderate to severe underweight (WAZ < −2) versus not (WAZ ≥ −2) and severe underweight (WAZ < −3) versus not (WAZ ≥ −3). The two underweight subgroups are compared with the not-underweight subgroup separately, and children in the severe underweight group will also belong to the moderate to severe underweight group. Children with a WAZ of <−6 or >5 were removed as outliers following WHO recommendations.^22^ Children who had weight measured at more than one round were only included once, at the first instance of weight measurement, which could occur at any phase of the study as this was a rolling census.

### Sample size and statistical methods

The sample size for this subgroup analysis is fixed and was based on the primary mortality trial. Two comparisons are included here, azithromycin 1–59 month versus placebo and azithromycin 1–11 month versus placebo. The trial had 80% power to detect a 10% relative reduction in mortality rates when comparing the azithromycin 1–59 month arm to the placebo arm in the primary analysis. When comparing the azithromycin 1–11 month arm to the placebo arm, the trial had 80% power to detect a 19% relative reduction. Alpha was set to 0.05 for both comparisons.

Analyses were conducted in R.^23^ Descriptive characteristics at the community level including age, sex and WAZ were summarised by arm and reported as frequency and percentage for categorical variables and mean and SD for numeric variables. Mortality rates were calculated for each subgroup in each arm as deaths per 1000 person-years, and 95% CIs were constructed using bootstrap resampling with 1000 replicates.

Poisson regression was used to estimate incidence rate ratios (IRRs) comparing the azithromycin 1–59 month arm to the placebo arm and the azithromycin 1–11 month arm to the placebo arm. The number of deaths in a community was the outcome, community-level person-time at risk was included as an offset and models included terms for treatment arm and for the interaction between underweight status and treatment. The adaptive allocation of the trial was accounted for by including it as an indicator variable. G-computation was used to estimate incidence rate differences (IRDs) from the Poisson model. The fitted model was used to predict the number of deaths under each treatment arm for each community at each underweight subgroup level. Mortality rates were then computed using the predicted number of deaths and the observed person-time in each cluster. Using these mortality rates, an IRD for each subgroup was calculated within each comparison. For the IRR and IRD, we estimated 95% confidence with a bootstrap, resampling communities with replacement (10 000 replicates).

Effect modification was evaluated on the multiplicative and additive scale using interaction contrasts.^24^ The multiplicative interaction contrast was determined by using the relevant interaction coefficient in the fitted Poisson model. To calculate the additive interaction contrast, the difference in IRDs was determined by subtracting the sum of the effects in the singly exposed groups from the effect in the doubly exposed group. An additive interaction contrast of greater than 0 signifies that the combined effect of azithromycin treatment and not being classified as underweight exceeds the sum of the individual effects of each independently. A multiplicative interaction contrast of greater than 1 indicates that the combined effect of azithromycin treatment and not being classified as underweight is greater than the product of each individual effect when considered independently. Permutation p values for the interaction contrasts are reported. An exploratory analysis examined the two regions separately using similar methods, though an additional interaction analysis for region was not conducted given the reduced power when stratified further.

### Patient and public involvement statement

Neither patients nor the public were involved in the design of this study. However, the Niger Ministry of Health contributed to the study’s design. Additionally, the Niger Ministry of Health, CSI leaders, community health workers and community leaders were engaged in the recruitment, implementation and dissemination phases of the study.

## Results

**Figure 1:**
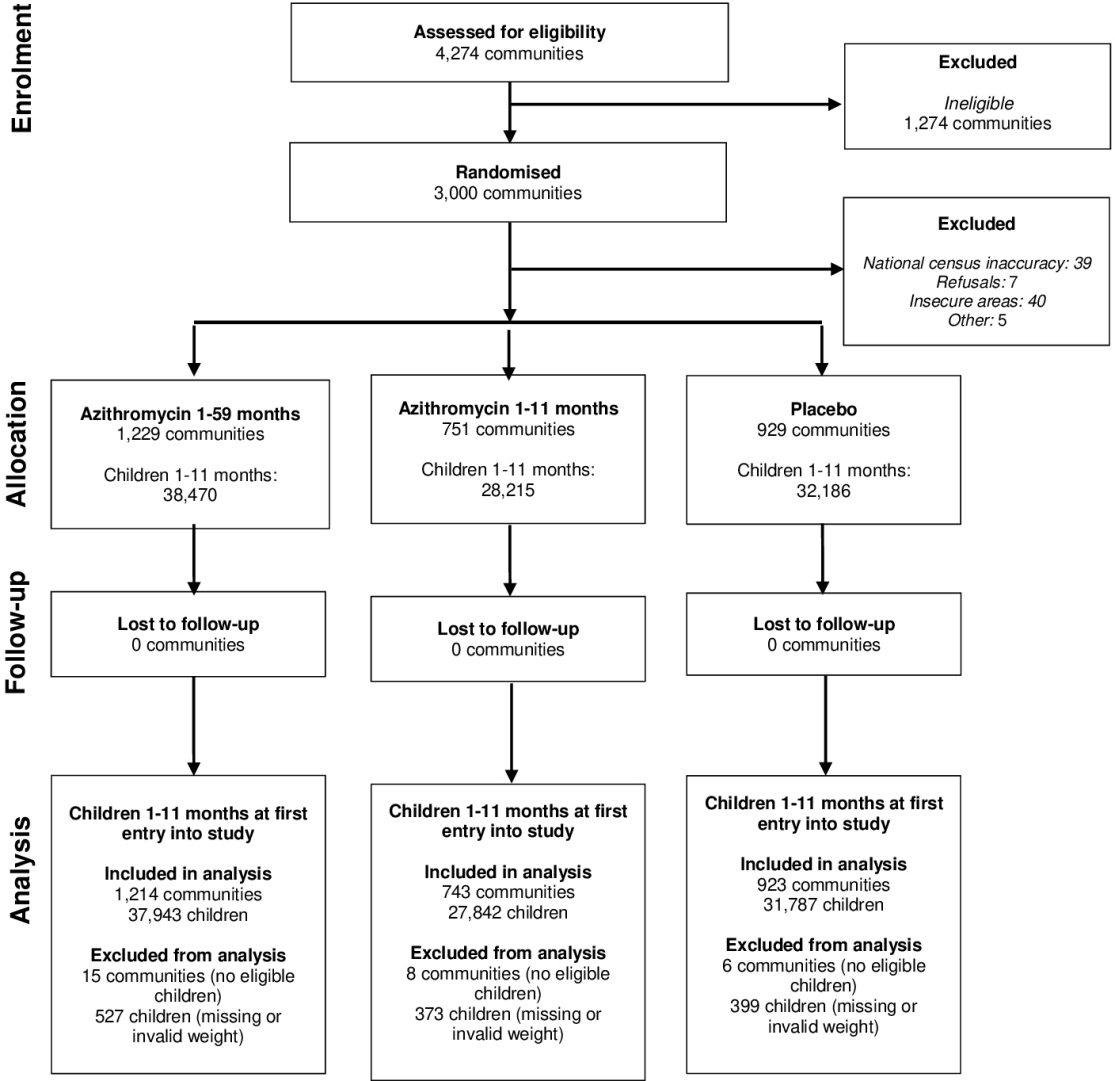
Consolidated Standards of Reporting Trials participant flow diagram.

The trial occurred between November 2020 and July 2023 with a total of 3000 communities enrolled with 98 968 children 1–11 months old participating over the course of the 2-year trial (figure 1). There were 2880 communities and 97 572 children 1–11 months old included in this analysis who were eligible for treatment and weighed during at least one round. 91 communities were excluded from the trial after randomisation due to national census inaccuracies. Communities were excluded from this analysis if they had accumulated no person-time for children 1–11 months old (26 communities) or if they had no children 1–11 months old with weights measured (three communities). 1076 children with missing weight were excluded from the analysis. 320 children were excluded from the analysis for having a WAZ <−6 or >5, as per WHO guidelines.^22^

Demographic statistics were similar among arms (table 1). At baseline, community-level mean WAZ was −1.16 (SD=0.58) in the azithromycin 1–59 month arm, −1.08 (SD=0.52) in the azithromycin 1–11 month arm and −1.09 (SD=0.56) in the placebo arm. Mean community proportion of moderate to severe underweight was 26.6% (SD=16.8%) in the azithromycin 1–59 month arm, 25.2% (SD=14.5%) in the azithromycin 1–11 month arm, 25.8% (SD=15.0%) in the azithromycin 1–59 month arm, 9.5% (SD=9.7%) in the azithromycin 1–11 month arm and 10.1% (SD=10.5%) in the placebo arm. Of the included children, there were 334 deaths and 18 413 person-years recorded in the azithromycin 1–59 month arm, 313 deaths and 13 935 person-years recorded in the azithromycin 1–11 month arm and 374 deaths and 15 603 person-years recorded in the placebo arm (table 2). Mortality rates were higher in underweight subgroups in all arms, particularly in the severe underweight subgroup (table 2).

**Table 1:** Community-level descriptive characteristics by arm of children at their first census

	Azithromycin 1–11 m	Azithromycin 1–59 m	Placebo	Overall
Age, mean (SD)	5.6 (0.8)	5.6 (0.9)	5.6 (0.8)	5.6 (0.9)
Percent female participants, mean (SD)	50.1% (11.6%)	50.1% (14.7%)	49.4% (13.2%)	49.9% (13.5%)
Mean child per community, mean (SD)	37.5 (30.4)	31.3 (26.6)	34.4 (26.9)	33.8 (27.8)
WAZ, mean (SD)	−1.08 (0.52)	−1.16 (0.58)	−1.09 (0.56)	−1.12 (0.56)
Proportion by underweight status, mean (SD)				
Moderate to severe (WAZ <−2)	25.2% (14.5%)	26.6% (16.8%)	25.8% (15.0%)	26.0% (15.7%)
Severe (WAZ <−3)	9.5% (9.7%)	10.8% (11.3%)	10.1% (10.5%)	10.2% (10.7%)

WAZweight-for-age z-score

**Table 2:** Mortality rate, deaths and person years stratified by subgroup and treatment group

Category	Azithromycin 1–59 m		Azithromycin 1–11 m		Placebo	
	Deaths,person-years	Mortality rate(95% CI)	Deaths,person-years	Mortality rate(95% CI)	Deaths,person-years	Mortality rate(95% CI)
Overall*	334, 18 413	18.1 (15.9, 20.1)	313, 13 935	22.5 (20.0, 25.2)	374, 15 603	24.0 (21.4, 26.6)
WAZ category, moderate to severe						
≥−2	211, 13 909	15.2 (13.1, 17.2)	212, 10 660	19.9 (17.3, 23.0)	242, 11 821	20.5 (18.4, 23.5)
<−2	123, 4504	27.3 (21.8, 32.2)	101, 3275	30.8 (25.2, 36.5)	132, 3781	34.9 (29.3, 40.9)
WAZ category, severe						
≥−3	268, 16 610	16.1 (13.9, 18.1)	269, 12 726	21.2 (18.8, 23.9)	305, 14 150	21.6 (19.5, 24.2)
<−3	66, 1803	36.6 (26.9, 44.7)	44, 1209	36.4 (26.8, 46.4)	69, 1453	47.5 (37.1, 58.4)

* Results will differ slightly from the main trial results since this subgroup analysis uses a different subpopulation.

WAZweight-for-age z-score

### Azithromycin 1-59 months versus placebo

**Figure 2:**
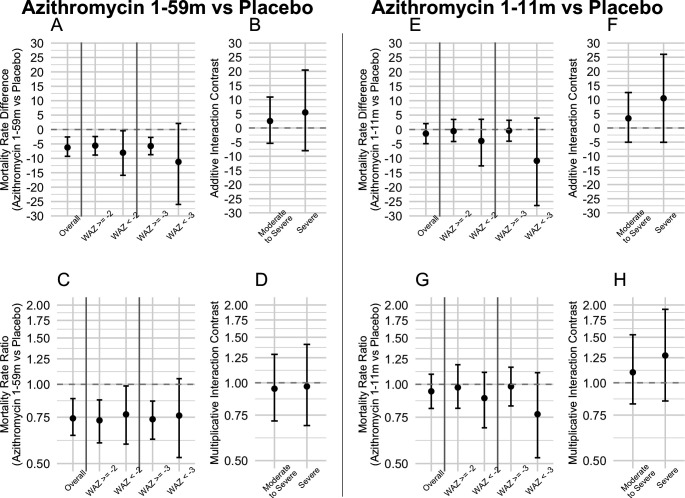
Incidence rate ratios, incidence rate differences and interaction contrasts within each arm and subgroup. (A) Incidence rate difference in the comparison of azithromycin 1-59 month arm versus placebo arm within each subgroup. (B) Additive interaction contrasts within each subgroup in the azithromycin 1-59 month arm versus placebo arm comparison. (C) Incidence rate ratio in the comparison of azithromycin 1-59 month arm versus placebo arm within each subgroup. (D) Multiplicative interaction contrasts within each subgroup in the azithromycin 1-59 month arm versus placebo arm comparison. (E) Incidence rate difference in the comparison of azithromycin 1-11 month arm versus placebo arm within each subgroup. (F) Additive interaction contrasts within each subgroup in the azithromycin 1-11 month arm versus placebo arm comparison. (G) Incidence rate ratio in the comparison of azithromycin 1-11 month arm versus placebo arm within each subgroup. (H) Multiplicative interaction contrasts within each subgroup in the azithromycin 1-11 month arm versus placebo arm comparison.

The azithromycin 1–59 month arm had lower mortality rates compared with the placebo arm overall and by subgroup (table 2). The moderate to severe subgroup had a 23% (95% CI 1% to 41%) reduction in mortality and an IRD of 8.0 (95% CI 0.4 to 15.9) fewer deaths per 1000 person-years in the azithromycin arm, while the severe underweight subgroup had a 24% (95% CI −5% to 47%) reduction in mortality and an IRD of 11.2 (95% CI −2.1 to 26.0) fewer deaths per 1000 person-years in the azithromycin arm (figure 2, online supplemental table 1). No interaction contrasts on the additive scale nor multiplicative scale were statistically significant for either subgroup (figure 2, online supplemental table 1). When analysed separately by region, in Dosso, the effect of azithromycin in the severely underweight group was more pronounced compared with that in Tahoua (online supplemental figure 1 and table 2). Dosso saw an IRD of 18.6 (95% CI −0.5 to 36.4) fewer deaths per 1000 person-years in the azithromycin arm among those severely underweight.

### Azithromycin 1-11 month versus placebo

In comparisons of the azithromycin 1–11 month arm versus placebo, the moderate to severe subgroup had an 11% (95% CI −11% to 32%) reduction in mortality and IRD of 4.0 (95% CI −3.6 to 12.6) fewer deaths per 1000 person-years in the azithromycin arm, while the severe underweight subgroup had a 23% (95% CI −11% to 47%) reduction in mortality and IRD of 10.9 (95% CI −4.0 to 26.4) fewer deaths per 1000 person-years in the azithromycin arm (figure 2, online supplemental table 1). No interaction contrasts on the additive scale nor multiplicative scale were statistically significant for either subgroup (figure 2, online supplemental table 1). When analysed separately by region, in Dosso, the effect of azithromycin in the severely underweight group was again more pronounced compared with that in Tahoua (online supplemental figure 2 and table 3). Dosso saw an IRD of 12.2 (95% CI −5.4 to 31.9) fewer deaths per 1000 person-years in the azithromycin arm among those severely underweight.

## Discussion

This subgroup analysis of the AVENIR trial in Niger evaluated the effect of biannual azithromycin distribution on rates of infant mortality by moderate and severe underweight status. Overall, the AVENIR trial found a 14% reduction in child mortality among children 1–59 months old after biannual azithromycin distribution but was unable to detect a difference in the comparison of communities of the biannual azithromycin 1–11 month and placebo arms.^3^ The analysis described here found higher mortality rates in both moderate to severe and severe underweight subgroups compared with non-underweight groups, which is consistent with previous literature.^1225^,^27^ Biannual azithromycin significantly reduced mortality rates in communities in the 1–59 month arm versus placebo in all subgroups, with larger reductions observed in children with both moderate to severe and severe underweight, particularly in the Dosso region, which may have a higher burden of mortality and infectious diseases associated with rainfall patterns. While the azithromycin 1–11 month arm showed a non-significant 6% reduction in infant mortality overall, the results suggest that children with severe malnutrition after biannual azithromycin distribution may have experienced an effect of the intervention, although we were not powered to detect this interaction. This reduction in infant mortality was especially pronounced in the Dosso region. Our results here mirror those from the primary analysis, which showed a significant reduction of child mortality in the 1–59 month arm and placebo comparison but did not find a significant reduction for the 1–11 month arm and placebo comparison.^3^ Although several analyses suggested the potential for interaction in severely underweight subgroups, we were unable to demonstrate underweight status as an effect modifier in any of the analyses presented here.

Previous research has studied the impact of antibiotic use in malnourished children on mortality and shown mixed results. When looking at individual-level treatment of children with severe acute malnutrition, one study found evidence for the association of antibiotics and the improvement in both malnutrition and mortality rates.^14^ However, another study was unable to demonstrate this improvement.^15^ The MORDOR trial showed higher mortality rates among malnourished children and suggested larger mortality reductions among underweight subgroups with community-level treatment but was unable to demonstrate underweight status as an effect modifier with treatment, similar to the present findings.^18^ The CHAT trial in Burkina Faso found a 51% reduction in the mortality of children 6–59 months old with acute malnutrition receiving azithromycin compared with placebo.^28^ Further, antibiotics are frequently used outside of this trial for children who are underweight for the treatment of severe acute malnourishment and other illnesses.^29^ It is possible that the use of other antibiotics could attenuate the effect of azithromycin for children in underweight subgroups. Together these results suggest the potential for larger effects of azithromycin MDA in underweight children, but larger studies would be required to detect interaction effects with a rare event like mortality.

Out of concern for antibiotic resistance, there have been calls to restrict the eligible population targeted for azithromycin MDA to limit antibiotic use.^30^ The mass distribution of antibiotics introduces selection for AMR not only to individuals receiving the treatment but to all members of the community.^31^ During the MORDOR trial, a higher prevalence of resistance to macrolides was detected in communities receiving azithromycin compared with placebo at the 24-, 36-, 48- and 60-month time points.^8 9 11^ Further, a non-significant 3.4% increase in macrolide resistance was found in an untreated population of children 7–12 years old at the 24-month time point, which indicates there may be spillover of AMR to untreated populations.^32^ Targeting specific groups at greater risk of mortality may lead to a decreased burden of AMR in the long term. At the same time, the main AVENIR trial suggests that community-level benefits of this intervention are achieved by treating the entire 1–59 month age group, due to the presence of both direct and indirect effects.^3^ Restricting eligibility to smaller high-risk subgroups like 1–11 months of age or underweight groups may eliminate the community-level effect and would reduce the overall numbers of lives saved.

There were a few limitations to this analysis. Although much larger than prior studies, this analysis was still underpowered to detect differences among subgroups given the focus on 1–11 month mortality, which represents a small subgroup and a rare outcome. While underweight status has been identified as an indicator of mortality risk, trial results could have been bolstered by including other indicators of malnutrition such as wasting and stunting. However, the required anthropometric measurements were not included in data collection, which had to be limited given the scale of the study.^26^ The inclusion restriction of rural and peri-urban small communities in Niger may limit generalisability to larger urban communities. Further, this trial only captured data for children 1–11 months old, and the underweight status of children 12–59 months old was not included in this analysis. It is possible that these age groups represent different risk patterns. Strengths include the systematic data collection of weight for all children aged 1–11 months. The cluster-randomised design of the AVENIR trial precluded bias in estimation of mortality effects, and inclusion of two regions in Niger led to greater generalisability of the results.

The AVENIR trial found a high prevalence of underweight and increased mortality rates among subgroups of children with underweight status across all arms. Although analyses suggest the potential for stronger effects in more severe underweight subgroups, we were unable to demonstrate underweight status as an effect modifier. In fact, azithromycin MDA to children 1–59 months old reduced mortality in all subgroups, and, especially as the majority of lives would be saved by treating all subgroups, our results do not support restricting eligibility for this intervention.

## supplementary material

10.1136/bmjopen-2024-097916 online supplemental file 1

## Data Availability

Data are available in a public, open access repository.
