# Supplementary material for: Mass distribution of azithromycin and child mortality among underweight infants in rural Niger: a subgroup analysis of the AVENIR cluster-randomised trial
Source: BMJ Open. 2025 Mar 27;15(3):e097916. doi: 10.1136/bmjopen-2024-097916 (PMC11956350; doi:10.1136/bmjopen-2024-097916)
Supplement: online supplemental file 1 [file bmjopen-15-3-s001.docx]

**Supplementary Material**

**Supplemental Table 1.** Incidence rate ratios, incidence rate differences, and interaction contrasts and their *P*-values by arm within each subgroup in the comparison of the azithromycin 1-59 month vs placebo arms and azithromycin 1-11 month vs placebo arms.

| Arm Comparison | Subgroup | Number of children | | IRR (95% CI) | Multiplicative interaction contrast (95% CI), *P*-value | IRD (95% CI) | Additive interaction contrast (95% CI), *P*-value |
| --- | --- | --- | --- | --- | --- | --- | --- |
|  |  | *Treatment Arm* | *Placebo Arm* |  |  |  |  |
| 1-59 month versus placebo | | | | | | | |
|  | Overall | 37,943 | 31,787 | 0.74  (0.64 to 0.88) |  | -6.2  (-9.3 to -2.6) |  |
|  | WAZ category,  Moderate to severe |  |  |  | 0.95  (0.71 to 1.29)  *P* = 0.74 |  | 2.4  (-5.5 to 10.9)  *P* = 0.55 |
|  | ≥ -2 | 28,490 | 23,960 | 0.73  (0.60 to 0.87) |  | -5.6  (-8.9 to -2.43) |  |
|  | < -2 | 9,453 | 7,827 | 0.77  (0.59 to 0.99) |  | -8.0  (-15.9 to -0.4) |  |
|  | WAZ category,  Severe |  |  |  | 0.97  (0.68 to 1.41)  *P* = 0.86 |  | 5.5  (-8.0 to 20.5)  *P* = 0.49 |
|  | ≥ -3 | 34,147 | 28,754 | 0.74  (0.62 to 0.86) |  | -5.7  (-8.8 to -2.7) |  |
|  | < -3 | 3,796 | 3,033 | 0.76  (0.53 to 1.05) |  | -11.2  (-26.0 to 2.1) |  |
| 1-11 month versus placebo | | | | | | | |
|  | Overall | 27,842 | 31,787 | 0.94  (0.81 to 1.09) |  | -1.4  (-4.9 to 2.1) |  |
|  | WAZ category,  Moderate to severe |  |  |  | 1.10  (0.83 to 1.53)  *P* = 0.57 |  | 3.4  (-5.0 to 12.5)  *P* = 0.48 |
|  | ≥ -2 | 21,208 | 23,960 | 0.97  (0.81 to 1.19) |  | -0.6  (-4.2 to 3.5) |  |
|  | < -2 | 6,634 | 7,827 | 0.89  (0.68 to 1.11) |  | -4.0  (-12.6 to 3.6) |  |
|  | WAZ category,  Severe |  |  |  | 1.27  (0.85 to 1.92)  *P* = 0.24 |  | 10.5  (-5.1 to 26.0)  *P* = 0.20 |
|  | ≥ -3 | 25,385 | 28,754 | 0.98  (0.83 to 1.16) |  | -0.4  (-4.0 to 3.2) |  |
|  | < -3 | 2,457 | 3,033 | 0.77  (0.53 to 1.11) |  | -10.9  (-26.4 to 4.0) |  |

**Supplemental Table 2.** Incidence rate ratios, incidence rate differences, and interaction contrasts and their *P*-values by region within each subgroup in the comparison of the azithromycin 1-59 month vs placebo arms.

| Region | Subgroup | Number of children | | IRR (95% CI) | Multiplicative interaction contrast (95% CI), *P*-value | IRD (95% CI) | Additive interaction contrast (95% CI), *P*-value |
| --- | --- | --- | --- | --- | --- | --- | --- |
|  |  | *Azithromycin*  *1-59 Arm* | *Placebo*  *Arm* |  |  |  |  |
| Dosso | | | | | | | |
|  | Overall | 21,997 | 20,884 | 0.71  (0.59 to 0.87) |  | -6.7  (-10.1 to -2.6) |  |
|  | WAZ category,  Moderate to severe |  |  |  | 0.98  (0.64 to 1.46)  *P* = 0.92 |  | 3.3  (-7.4 to 13.9)  *P* = 0.53 |
|  | ≥ -2 | 17,085 | 16,167 | 0.70  (0.55 to 0.87) |  | -5.9  (-9.7 to -2.2) |  |
|  | < -2 | 4,912 | 4,717 | 0.72  (0.49 to 1.02) |  | -9.2  (-20.0 to 0.6) |  |
|  | WAZ category,  Severe |  |  |  | 1.19  (0.68 to 2.06)  *P* = 0.53 |  | 12.9  (-7.2 to 30.3)  *P* = 0.17 |
|  | ≥ -3 | 20,112 | 19,193 | 0.72  (0.57 to 0.88) |  | -5.7  (-9.5 to -2.2) |  |
|  | < -3 | 1,885 | 1,691 | 0.61  (0.37 to 1.01) |  | -18.6  (-36.4 to 0.5) |  |
| Tahoua | | | | | | | |
|  | Overall | 15,946 | 10,903 | 0.80  (0.62 to 1.04) |  | -5.4  (-11.4 to 1.0) |  |
|  | WAZ category,  Moderate to severe |  |  |  | 0.95  (0.57 to 1.53)  *P* = 0.82 |  | 1.9  (-13.2 to 16.8)  *P* = 0.76 |
|  | ≥ -2 | 11,405 | 7,793 | 0.78  (0.57 to 1.10) |  | -4.8  (-11.2 to 1.8) |  |
|  | < -2 | 4,541 | 3,110 | 0.83  (0.55 to 1.22) |  | -6.7  (-20.9 to 6.7) |  |
|  | WAZ category,  Severe |  |  |  | 0.80  (0.46 to 1.41)  *P* = 0.41 |  | -3.6  (-26.3 to 20.8)  *P* = 0.76 |
|  | ≥ -3 | 14,035 | 9,561 | 0.76  (0.58 to 1.02) |  | -5.7  (-11.5 to 0.4) |  |
|  | < -3 | 1,911 | 1,342 | 0.96  (0.57 to 1.56) |  | -2.1  (-26.5 to 19.3) |  |

**Supplemental Table 3.** Incidence rate ratios, incidence rate differences, and interaction contrasts and their *P*-values by region within each subgroup in the comparison of the azithromycin 1-11 month vs placebo arms.

| Region | Subgroup | Number of children | | IRR (95% CI) | Multiplicative interaction contrast (95% CI), *P*-value | IRD (95% CI) | Additive interaction contrast (95% CI), *P*-value |
| --- | --- | --- | --- | --- | --- | --- | --- |
|  |  | *Azithromycin 1-11 Arm* | *Placebo*  *Arm* |  |  |  |  |
| Dosso | | | | | | | |
|  | Overall | 20,396 | 20,884 | 1.03  (0.85 to 1.24) |  | 0.7  (-3.8 to 4.9) |  |
|  | WAZ category,  Moderate to severe |  |  |  | 1.15  (0.78 to 1.71)  *P* = 0.49 |  | 3.5  (-7.1 to 14.8)  *P* = 0.51 |
|  | ≥ -2 | 15,774 | 16,167 | 1.07  (0.86 to 1.34) |  | 1.4  (-3.1 to 5.9) |  |
|  | < -2 | 4,622 | 4,717 | 0.94  (0.67 to 1.27) |  | -2.1  (-12.7 to 7.6) |  |
|  | WAZ category,  Severe |  |  |  | 1.46  (0.92 to 2.43)  *P* = 0.14 |  | 14.0  (-4.4 to 33.7)  *P* = 0.17 |
|  | ≥ -3 | 18,743 | 19,193 | 1.09  (0.88 to 1.33) |  | 1.8  (-2.5 to 6.0) |  |
|  | < -3 | 1,653 | 1,691 | 0.74  (0.46 to 1.14) |  | -12.2  (-31.9 to 5.4) |  |
| Tahoua | | | | | | | |
|  | Overall | 7,446 | 10,903 | 0.73  (0.53 to 0.97) |  | -7.3  (-14.1 to -0.7) |  |
|  | WAZ category,  Moderate to severe |  |  |  | 0.85  (0.47 to 1.58)  *P* = 0.60 |  | 0.5  (-16.9 to 17.7)  *P* = 0.97 |
|  | ≥ -2 | 5,434 | 7,793 | 0.69  (0.46 to 1.04) |  | -6.9  (-13.8 to 0.7) |  |
|  | < -2 | 2,012 | 3,110 | 0.81  (0.52 to 1.27) |  | -7.3  (-22.0 to 9.1) |  |
|  | WAZ category,  Severe |  |  |  | 0.87  (0.43 to 1.76)  *P* = 0.68 |  | 1.3  (-25.7 to 27.2)  *P* = 0.93 |
|  | ≥ -3 | 6,642 | 9,561 | 0.72  (0.50 to 1.02) |  | -6.7  (-13.5 to 0.5) |  |
|  | < -3 | 804 | 1,342 | 0.83  (0.42 to 1.49) |  | -8.0  (-33.6 to 18.9) |  |

**Supplemental Figure 1.** Incidence rate ratios, incidence rate differences, and interaction contrasts within each subgroup in the comparison of the azithromycin 1-59 month vs placebo arms.


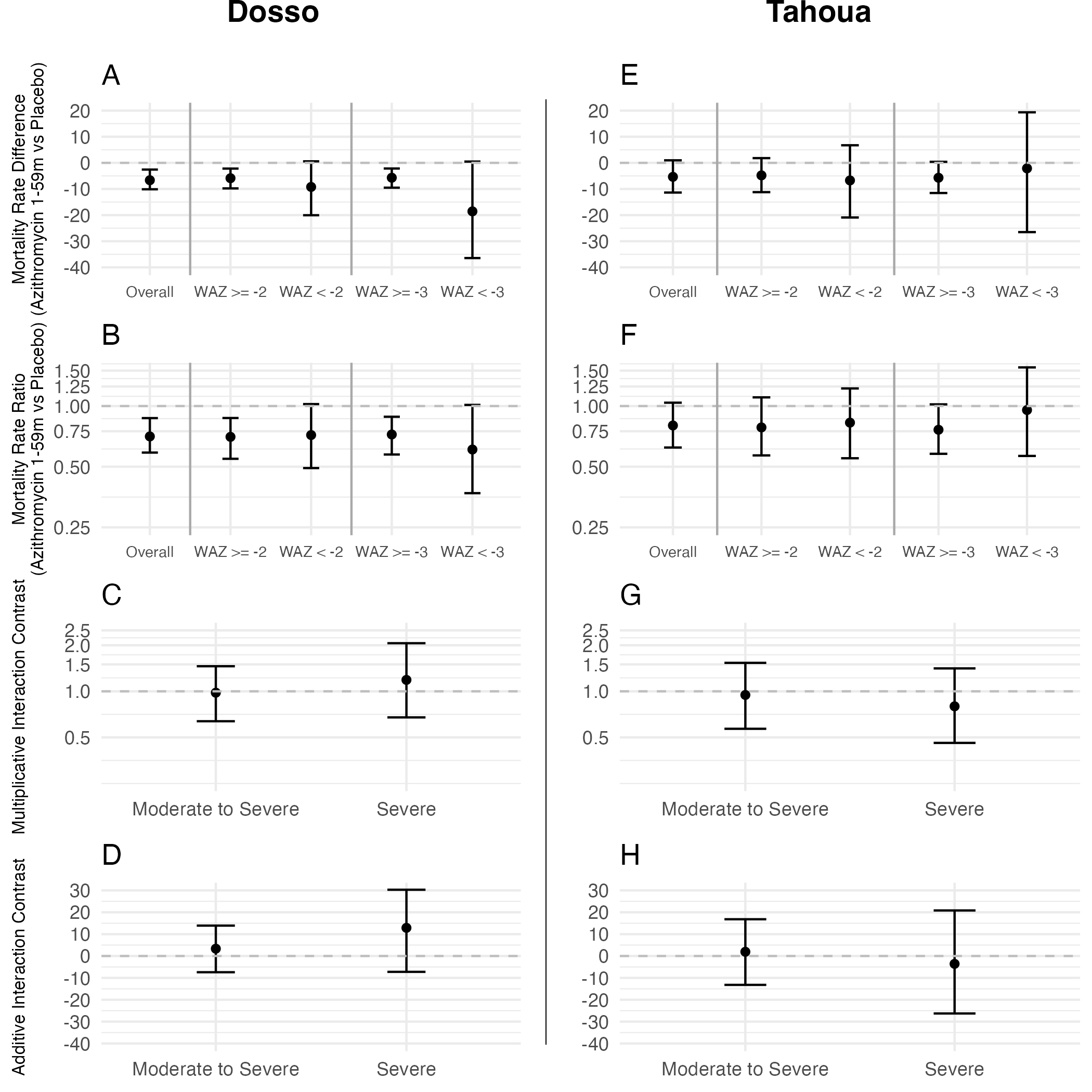


***A.** Incidence rate difference within each subgroup in Dosso region. **B.** Incidence rate ratio within each subgroup in Dosso region. **C.** Multiplicative interaction contrasts within each subgroup in the Dosso region. **D.** Additive interaction contrasts within each subgroup in the Dosso region. **E.** Incidence rate difference within each subgroup in Tahoua region. **F.** Incidence rate ratio within each subgroup in Tahoua region. **G.** Multiplicative interaction contrasts within each subgroup in the Tahoua region. **H.** Additive interaction contrasts within each subgroup in the Tahoua region.

**Supplemental Figure 2.** Incidence rate ratios, incidence rate differences, and interaction contrasts within each subgroup in the comparison of the azithromycin 1-11 month vs placebo arms.


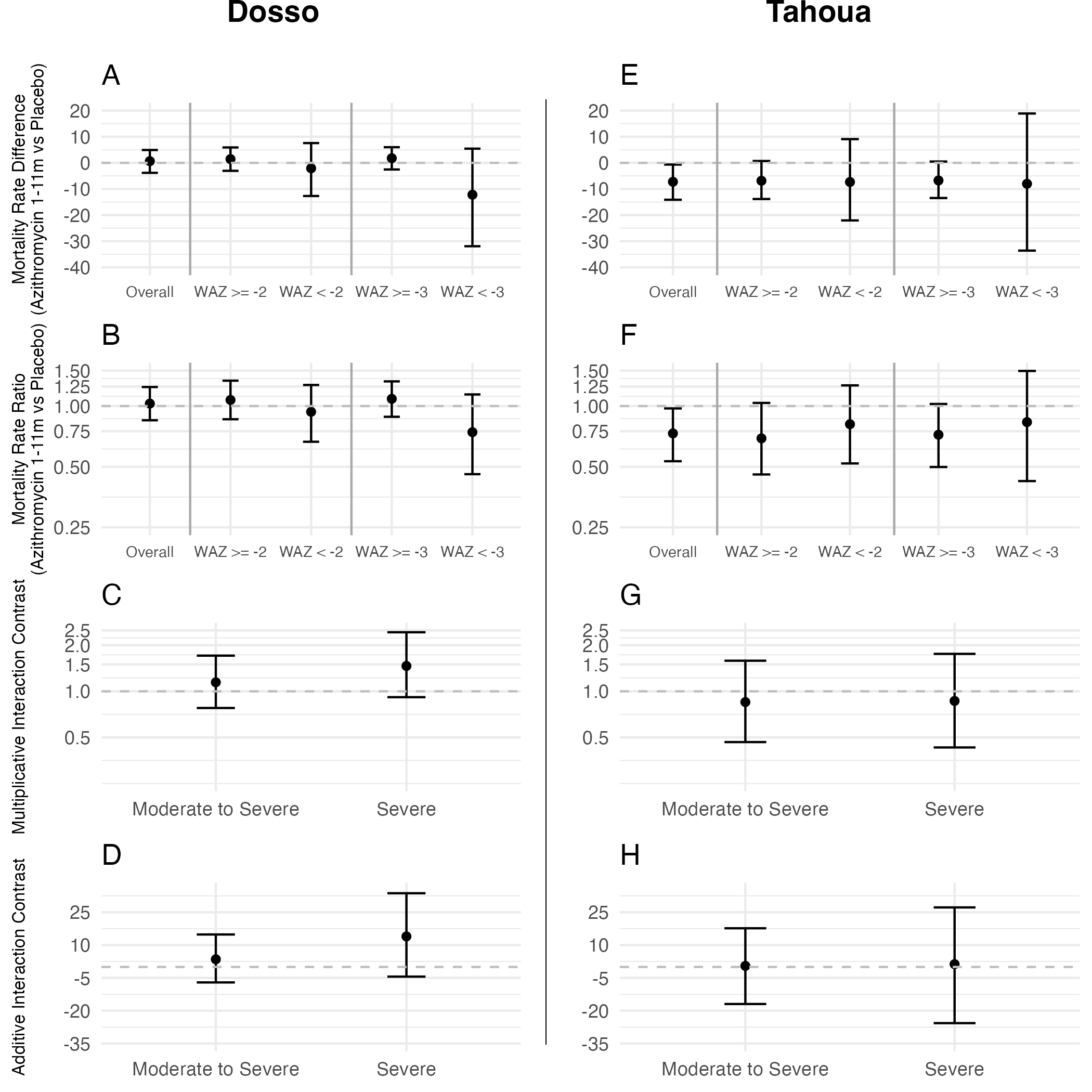


***A.** Incidence rate difference within each subgroup in Dosso region. **B.** Incidence rate ratio within each subgroup in Dosso region. **C.** Multiplicative interaction contrasts within each subgroup in the Dosso region. **D.** Additive interaction contrasts within each subgroup in the Dosso region. **E.** Incidence rate difference within each subgroup in Tahoua region. **F.** Incidence rate ratio within each subgroup in Tahoua region. **G.** Multiplicative interaction contrasts within each subgroup in the Tahoua region. **H.** Additive interaction contrasts within each subgroup in the Tahoua region.
