# Supplementary material for: Mass distribution of azithromycin and child mortality among underweight infants in rural Niger: a subgroup analysis of the AVENIR cluster-randomised trial
Source: BMJ Open. 2025 Mar 27;15(3):e097916. doi: 10.1136/bmjopen-2024-097916 (PMC11956350; doi:10.1136/bmjopen-2024-097916)
Supplement: Supplementary file 2 [file bmjopen-2024-097916.reviewer_comments.pdf]

# PEER REVIEW HISTORY

BMJ Open publishes all reviews undertaken for accepted manuscripts. Reviewers are asked to complete a checklist review form and are provided with free text boxes to elaborate on their assessment. These free text comments are reproduced below.

## ARTICLE DETAILS

### Title (Provisional)

Mass distribution of azithromycin and child mortality among underweight infants in rural Niger: a subgroup analysis of the AVENIR cluster-randomized trial

### Authors

Peterson, Brittany; Arzika, Ahmed; Maliki, Ramatou; Abdou, Amza; Aichatou, Bawa; Bello, Ismael M; Beidi, Diallo; Galo, Nasser; Harouna, Nasser; Karamba, Alio M; Mahamadou, Sani; Abarchi, Moustapha; Ibrahim, Almou; Lebas, Elodie; Liu, Zijun; Brandt, Carolyn; Colby, Emily; Oldenburg, Catherine E; Porco, Travis C.; Arnold, Benjamin; Lietman, Thomas M.; O'Brien, Kieran Sunanda

---

## VERSION 1 - REVIEW

---

| | |
|---|---|
| **Reviewer** | **1** |
| **Name** | **von Seidlein, Lorenz** |
| **Affiliation** | **Menzies School of Health Research** |
| **Date** | **04-Jan-2025** |
| **COI** | **None** |

---

Review

This paper provides valuable information and will be of interest to readers working on nutrition and interventions in pediatric population in LMICs.

I find the conclusions quite satisfactory. There is a trend for increased protection in the most undernourished children, yet the investigators conclude rightly (in my opinion) that all undernourished participants should receive the intervention. I think this particularly appropriate as moderately undernourished children will benefit from the intervention.

Methods

The authors write:

L142 “ communities randomized to three arms: azithromycin to children 1-59 months, azithromycin to children 1-11 months with placebo to children 12-59 months, and placebo to all children aged 1-59 months.” I find this confusing because in the sentence they recognize not three but four arms:

1. azithromycin to children 1-59 months,

2. azithromycin to children 1-11 months with

3. placebo to 144 children 12-59 months, and

4. placebo to all children aged 1-59 months.

The readers will appreciate it if the authors harmonize the numbering 3 versus 4 and describe only 3 arms as you do in the consort chart?

L165 “Weight was captured for all children 1-11 months that participated in treatment.” considering that the weight measurement is the topic of the paper a single sentence for the description of the process may be unsatisfactory for the readers? Please describe the process in more detail.

• Who did the weighing of the participants?

• How were they trained.

• Was it is always the same investigator who measured the same child.

• Which scales were used for each age band?

L173 “The trial used a response-adaptive allocation that is detailed in the primary results paper.” Please describe what is a response-adaptive allocation in a sentence for readers unable or unwilling to look up your reference paper.

Table 2 the authors compare the children split into two age groups with all children receiving placebo combined. This is not informative. Please split the children receiving placebo in the same fashion you split the children receiving azithromycin to allow the reader to compare same with same.

Figure 2 please indicate what A, B, C, … stands for?

---

| | |
|---|---|
| **Reviewer** | **2** |
| **Name** | **Pickering, Harry** |
| **Affiliation** | **London School of Hygiene & Tropical Medicine** |
| **Date** | **10-Jan-2025** |
| **COI** | **None** |

---

This study assessed the impact of azithromycin MDA on mortality, stratified by underweight subgrouping. The impact of azithro on mortality was greatest in the severe underweight subgroups, but there was no significant interaction of subgrouping with mortality reductions.

No major criticisms, my main question would just be whether this analysis is a significant improvement over ref-18, as the authors say both are ultimately underpowered to look at this subgroup analysis robustly.

Minor points below…

Line 49: If there's space, it might be helpful if the abstract briefly mentioned that azithro has been shown to reduce all-cause mortality, to give some rationale for looking within the subgroups here.

Line 64: Confidence interval has three numbers.

Line 71: What does "the majority of lives" mean in this context? Is the point that the greatest benefit would be achieved treating all subgroups? Rephrase to clarify.

Line 111: I think it should be "lowers" instead of "lower".

Line 115: Not sure all these references, or the literature at-large, uniformly support this statement.

---

| | |
|---|---|
| **Reviewer** | **3** |
| **Name** | **Walson, Judd** |
| **Affiliation** | **University of Washington, Department of Global Health** |
| **Date** | **16-Jan-2025** |
| **COI** | **None** |

---

This is an interesting paper that presents important findings from a secondary analysis of a large cluster randomized trial of azithro to prevent child mortality. The authors have attempted to answer an important question related to the relative benefits among children with anthropometric deficits and have appropriately noted the limitations of the analysis. Unfortunately, as a secondary analysis, it is difficult to interpret the findings in a way that will influence policy - but the paper is valuable nonetheless.

A few questions/comments. below;

1) It was difficult to understand how the analysis considered the exposure (underweight) in relation to the outcome. I assume that only the most recent census weight measure was used? Was any attempt made to look at change in WAZ between census rounds as an exposure, this may be more useful? At the very least – time between measured weight and death should be included in the model.

2) It is likely that underweight children may have been more likely to have recently received other antibiotics, for illness or treatment of SAM, which could attenuate the impact of Azithro on this group.

3) May be important to differentiate those who are preterm/LBW vs. not in evaluating differential mortality benefit

4) Restrictive inclusion of rural/peri-rural small communities may serve to include a population that is not generalizable to entire populations within countries.

5) Note that while WHO does recommend removing children with a WAZ of < -6 or >5, children do reach these levels and these would obviously be the highest risk children.

6) More detail on weight assessment should be included – were weights measured more than once and averaged? How as QA/QC done?

7) Lack of WAZ in the older children 12-59 months is a limitation as underweight represents different risk patterns in this group than in the younger child.

8) Clarify why 3000 communities were included in the trial but only 2880 used for this analysis? From figure 1, it looks like these communities were excluded before allocation of the intervention – but the main trial reports on 3000 communities (is this correct)?

9) It appears that 320 children were excluded for WAZ <6 or >5 – how many children were excluded for missing weights overall and in the 2880 included communities specifically?

10) The larger magnitude of effect in mortality reduction among those who were moderately to severely underweight in the 1-59 month old group may represent a true difference due to the underlying risk factors for death in this population and the differences in exposure and access to care and antibiotics. This would be supported by the overall findings of the AVENIR trial which also showed significant impact when older children were included but not when restricted to treatment of only 1-11 months olds.

11) The authors speculate that the larger reductions in mortality observed in Dosso may be due to infectious diseases related to rainfall patterns. Are there data to support this? There are likely many features related to access, disease endemicity, poverty, etc that may differ between Dosso and other regions – has this been explored and described previously?

12) AMR is also linked to malnutrition (higher rates of AMR among children with malnutrition) – likely as a result of greater exposure to hospitalization and to antibiotics. Was this examined?

13) The number of deaths is obviously a function of the number of children included, and there were far more children included in the Azithro 1-59 month arm. Can you explain a bit more in the design why this arm was oversampled in the main trial if randomization was done 1:1:1 per the protocol?

---

| | |
|---|---|
| **Reviewer** | **4** |
| **Name** | **Dack, Kyle** |
| **Affiliation** | **University of Bristol, MRC Integrative Epidemiology Unit** |

| | |
|---|---|
| **Date** | **30-Jan-2025** |
| **COI** | **None** |

---

I do not find any major methodological or statistical issues in this manuscript – methods are described clearly, and results are accurately reported. My main concern is how the subgroups overlap – a child can be both "moderate to severe underweight" and "not severely underweight", or both "moderate to severe underweight" and "severely underweight". I think more explanation is needed to why these categories were used, rather than making non-overlapping categories. More care is also needed in parts of the results and discussion care so that readers do not misinterpret the subgroups as being unique.

Abstract

1. Line 53-54. The way this sentence is phrased gives the impression that all children in the study were either moderate-to-severely, or severely underweight, when in fact it also included children not underweight. I suggest replacing "categorizes" with "identify/flag".

2. Line 62. Hard to follow, suggest re-ordering to say "Incidence of death in children 1-59 months in those receiving azithromycin compared to placebo were…"

3. Line 70. MDA is undefined.

Introduction

4. Line 111. Lower should be "lowers"

Methods

5. The main trial paper may already report the full study timeline, but I think more basic details should also be reported here for transparency and to save readers time: how were participants recruited? When was the intervention provided – during the biannual visits?

6. Nutritional status is measured by proxy through children's age-adjusted weight z-score. When children were weighed more than once, status was determined by the first measurement. I imagine this means that most children are defined by weight at the first census visit – but when was this first visit?

7. Line 191. This seems to be four overlapping categories, rather than two. There are four categories: moderate-to-severe underweight, not moderate-to-severe underweight, severe underweight, and not severe underweight. Children with a WAZ between -2 and -3 will be in both the moderate-to-severe underweight and not severe underweight subgroups, children with WAZ of -4 will be classed as both moderate-to-severe and severely underweight.

8. It does not really make sense to me that someone in the severely underweight category will also be classed as moderate-to-severe. It would be more accurate to call the latter category "moderate or severe".

Why was this approach used rather than creating three non-overlapping groups? E.g. WAZ > -2, WAZ -2 to -3, and WAZ < -3 ? This would enable comparisons between moderate and severely underweight children which in the currentl design is restricted due to categories having non-independent samples.

Results

9. Line 255-257. Using the terminology “the moderate to severe subgroup” and “the severe underweight subgroup”, it currently reads that these are independent subgroups, which they are not. I think some form of rephrasing is needed throughout the paper to avoid giving readers the wrong impression, phrases such as “those either moderate or severely underweight” might help – this removes the words “the” and “subgroup” both of which imply unique membership.

10. Supplementary tables 1-3. Please add the N to these tables.

Discussion

11. Results are interpreted accurately, and limitations described. My only suggestion is the same as previous sections – I recommend clarifying the description of the subgroups so that readers do not misinterpret them as being unique. E.g. lines 286-287.

---

## VERSION 1 - AUTHOR RESPONSE

**Reviewer: 1**
**Dr. Lorenz von Seidlein, Menzies School of Health Research**
**Comments to the Author:**
**Review**

**This paper provides valuable information and will be of interest to readers working on nutrition and interventions in pediatric population in LMICs.**
**I find the conclusions quite satisfactory. There is a trend for increased protection in the most undernourished children, yet the investigators conclude rightly (in my opinion) that all undernourished participants should receive the intervention. I think this particularly appropriate as moderately undernourished children will benefit from the intervention.**

**Methods**

**The authors write:**
**L142 “ communities randomized to three arms: azithromycin to children 1-59 months, azithromycin to children 1-11 months with placebo to children 12-59 months, and placebo to all children aged 1-59 months.” I find this confusing because in the sentence they recognize not three but four arms:**
**1. azithromycin to children 1-59 months,**
**2. azithromycin to children 1-11 months with**

**3. placebo to 144 children 12-59 months, and**
**4. placebo to all children aged 1-59 months.**
**The readers will appreciate it if the authors harmonize the numbering 3 versus 4 and describe only 3 arms as you do in the consort chart?**

Thanks for pointing this out. There are 3 arms in this study. The 2nd arm includes azithromycin to children 1-11 months with placebo to children 12-59 months all in one community. Placebo is offered to children 12-59 months to maintain masking. We've updated wording where we state this in lines 156-158.

**L165 "Weight was captured for all children 1-11 months that participated in treatment." considering that the weight measurement is the topic of the paper a single sentence for the description of the process may be unsatisfactory for the readers? Please describe the process in more detail.**
**• Who did the weighing of the participants?**
**• How were they trained.**
**• Was it is always the same investigator who measured the same child.**
**• Which scales were used for each age band?**

We've added more details on the process including exact type of scale used, who did the weighing, and how it was entered, please see lines 178-182.

**L173 "The trial used a response-adaptive allocation that is detailed in the primary results paper." Please describe what is a response-adaptive allocation in a sentence for readers unable or unwilling to look up your reference paper.**

Thanks for this suggestion. We've added more details regarding the response-adaptive allocation in lines 197-200.

**Table 2 the authors compare the children split into two age groups with all children receiving placebo combined. This is not informative. Please split the children receiving placebo in the same fashion you split the children receiving azithromycin to allow the reader to compare same with same.**

The headers in table 2 indicate which arm the community is in. There are 3 arms which are described in lines 156-158. These do not indicate age groups included in the analysis, rather they indicate what age groups receive azithromycin in these arms. The only age group included in this analysis are children 1-11 months old.

**Figure 2 please indicate what A, B, C, ... stands for?**

We've included a footnote the indicate what the letters stand for.

**Reviewer: 2**
**Dr. Harry Pickering, London School of Hygiene & Tropical Medicine**
**Comments to the Author:**
**This study assessed the impact of azithromycin MDA on mortality, stratified by**

**underweight subgrouping. The impact of azithro on mortality was greatest in the severe underweight subgroups, but there was no significant interaction of subgrouping with mortality reductions.**
**No major criticisms, my main question would just be whether this analysis is a significant improvement over ref-18, as the authors say both are ultimately underpowered to look at this subgroup analysis robustly.**

Thanks for your comments. This analysis uses data from a trial (AVENIR) that followed the one in ref-18 (MORDOR). AVENIR updated protocols to include capturing weight for all children 1-11 months compared to only children who can't stand in MORDOR and included more communities in more regions of Niger which increased the sample size (described in lines 144-146).

**Minor points below...**
**Line 49: If there's space, it might be helpful if the abstract briefly mentioned that azithro has been shown to reduce all-cause mortality, to give some rationale for looking within the subgroups here.**

Thanks for the suggestion, we've added this information in the abstract, line 49.

**Line 64: Confidence interval has three numbers.**

Thanks for catching this, we've updated this. Now line 66.

**Line 71: What does "the majority of lives" mean in this context? Is the point that the greatest benefit would be achieved treating all subgroups? Rephrase to clarify.**

We've updated the wording here to state "especially as the number of lives saved would be highest by treating all subgroups", line 73.

**Line 111: I think it should be "lowers" instead of "lower".**

Thanks for catching this, we've updated the wording to state "leads to lower under-5 mortality rates", line 122.

**Line 115: Not sure all these references, or the literature at-large, uniformly support this statement.**

We agree the placement of these references may be misleading. We've updated the wording to be more clear about what these references show, lines 123-124.

**Reviewer: 3**
**Dr. Judd Walson, University of Washington**
**Comments to the Author:**
**This is an interesting paper that presents important findings from a secondary analysis of a large cluster randomized trial of azithro to prevent child mortality. The authors have attempted to answer an important question related to the relative**

**benefits among children with anthropometric deficits and have appropriately noted the limitations of the analysis. Unfortunately, as a secondary analysis, it is difficult to interpret the findings in a way that will influence policy - but the paper is valuable nonetheless.**
**A few questions/comments. below;**
**1) It was difficult to understand how the analysis considered the exposure (underweight) in relation to the outcome. I assume that only the most recent census weight measure was used? Was any attempt made to look at change in WAZ between census rounds as an exposure, this may be more useful? At the very least – time between measured weight and death should be included in the model.**

The analysis was done at the community level, with treatment arm being the exposure and mortality as the outcome. Each community was split into two groups: children with underweight status and children without underweight status. The first recorded weight of a child was used and children were only included once in the analysis dataset, see lines 266-228. This binary variable was used to determine presence of interaction between underweight status and treatment. Because this analysis was done at the community level we did not look at change in WAZ and were not able to include time between measured weight and death in the model.

**2) It is likely that underweight children may have been more likely to have recently received other antibiotics, for illness or treatment of SAM, which could attenuate the impact of Azithro on this group.**

This is a great point, we've added this information and cited the WHO guidelines for treatment of SAM, see lines 354-357.

**3) May be important to differentiate those who are preterm/LBW vs. not in evaluating differential mortality benefit**

Because we only treated children who were at least 1 month old we unfortunately do not have data regarding preterm or low birth weight for these children.

**4) Restrictive inclusion of rural/peri-rural small communities may serve to include a population that is not generalizable to entire populations within countries.**

Thanks for pointing this out. We've added this as a limitation to generalizability, see lines 380-381.

**5) Note that while WHO does recommend removing children with a WAZ of < -6 or >5, children do reach these levels and these would obviously be the highest risk children.**

We've excluded these children in order to follow WHO guidelines. We've rerun the analysis without this exclusion and the results did not change.

**6) More detail on weight assessment should be included – were weights measured more than once and averaged? How as QA/QC done?**

We've added more detail on the weight assessment, see lines 178-182.

**7) Lack of WAZ in the older children 12-59 months is a limitation as underweight represents different risk patterns in this group than in the younger child.**

Thanks for this explanation. We've added this as a limitation, see lines 381-383.

**8) Clarify why 3000 communities were included in the trial but only 2880 used for this analysis? From figure 1, it looks like these communities were excluded before allocation of the intervention – but the main trial reports on 3000 communities (is this correct)?**

The main trial excluded 91 communities post-randomization due to national census inaccuracies. We've added more details about exclusions of children and communities, see lines 276-281.

**9) It appears that 320 children were excluded for WAZ <6 or >5 – how many children were excluded for missing weights overall and in the 2880 included communities specifically?**

We've added more details about exclusions of children and communities, see lines 276-281.

**10) The larger magnitude of effect in mortality reduction among those who were moderately to severely underweight in the 1-59 month old group may represent a true difference due to the underlying risk factors for death in this population and the differences in exposure and access to care and antibiotics. This would be supported by the overall findings of the AVENIR trial which also showed significant impact when older children were included but not when restricted to treatment of only 1-11 months olds.**

Thanks for this comment. We've added clarification in the discussion that this effect was significant in the overall trial, see lines 339-341.

**11) The authors speculate that the larger reductions in mortality observed in Dosso may be due to infectious diseases related to rainfall patterns. Are there data to support this? There are likely many features related to access, disease endemicity, poverty, etc that may differ between Dosso and other regions – has this been explored and described previously?**

While we currently do not have data published to support this, only anecdotal evidence, we do a have a publication forthcoming regarding the seasonality of WAZ patterns and

relation to rainfall and temperature. This publication is under review and will hopefully be published around the same time as this current paper.

**12) AMR is also linked to malnutrition (higher rates of AMR among children with malnutrition) – likely as a result of greater exposure to hospitalization and to antibiotics. Was this examined?**

We did not include AMR endpoints in this analysis, but agree this would be interesting to examine and will consider it for the future.

**13) The number of deaths is obviously a function of the number of children included, and there were far more children included in the Azithro 1-59 month arm. Can you explain a bit more in the design why this arm was oversampled in the main trial if randomization was done 1:1:1 per the protocol?**

The main trial used a response-adaptive allocation which changed the treatment ratio at the 2nd and 3rd timepoint of enrollment to have a higher proportion of communities with the best outcome which in this case was the azithro 1-59 month arm. We've added more information regarding this, see lines 197-200.

**Reviewer: 4**
**Dr. Kyle Dack, University of Bristol**
**Comments to the Author:**
**I do not find any major methodological or statistical issues in this manuscript – methods are described clearly, and results are accurately reported. My main concern is how the subgroups overlap – a child can be both "moderate to severe underweight" and "not severely underweight", or both "moderate to severe underweight" and "severely underweight". I think more explanation is needed to why these categories were used, rather than making non-overlapping categories. More care is also needed in parts of the results and discussion care so that readers do not misinterpret the subgroups as being unique.**

**Abstract**
**1. Line 53-54. The way this sentence is phrased gives the impression that all children in the study were either moderate-to-severely, or severely underweight, when in fact it also included children not underweight. I suggest replacing "categorizes" with "identify/flag".**

Thanks for pointing out this confusion. We've updated the sentence to say "Weight-for-age z-score was used to categorize children into subgroups of either moderate to severe underweight or not and severe underweight or not."

**2. Line 62. Hard to follow, suggest re-ordering to say "Incidence of death in children 1-59 months in those receiving azithromycin compared to placebo were..."**

We’ve updated this sentence to be more clear saying “Incidence rate differences of deaths in children 1-11 months comparing communities receiving azithromycin to children 1-59 months of age to placebo were..”, line 63

**3. Line 70. MDA is undefined.**

Thanks for pointing out this omission, we’ve defined it now on line 72.

**Introduction**
**4. Line 111. Lower should be “lowers”**

We’ve updated this, now on line 122.

**Methods**
**5. The main trial paper may already report the full study timeline, but I think more basic details should also be reported here for transparency and to save readers time: how were participants recruited? When was the intervention provided – during the biannual visits?**

We’ve added more detail in the methods stating that all households in the community were approached for participation (line 173) and that treatment was provided at the time of census (line 176).

**6. Nutritional status is measured by proxy through children’s age-adjusted weight z-score. When children were weighed more than once, status was determined by the first measurement. I imagine this means that most children are defined by weight at the first census visit – but when was this first visit?**

Children could be recruited into the project at any phase of the study as this was a rolling census. So by first measurement we are referring to the individual’s first census and not necessarily the study’s first census. We’ve added clarification on line 227-228.

**7. Line 191. This seems to be four overlapping categories, rather than two. There are four categories: moderate-to-severe underweight, not moderate-to-severe underweight, severe underweight, and not severe underweight. Children with a WAZ between -2 and -3 will be in both the moderate-to-severe underweight and not severe underweight subgroups, children with WAZ of -4 will be classed as both moderate-to-severe and severely underweight.**

In this analysis the two subgroups are analyzed separately, so children may be included in one, both, or none of the underweight subgroups. The aim of this analysis was to see if either of the two cutoff points of WAZ would provide a statistically significant effect modification. We’ve added more details regarding the subgroups stating “The two subgroups are analyzed separately, and children in the severe underweight group will also belong to the moderate to severe underweight group” on lines 220-225.

**8. It does not really make sense to me that someone in the severely underweight**

**category will also be classed as moderate-to-severe. It would be more accurate to call the latter category “moderate or severe”.**
**Why was this approach used rather than creating three non-overlapping groups? E.g. WAZ > -2, WAZ -2 to -3, and WAZ < -3 ? This would enable comparisons between moderate and severely underweight children which in the currentl design is restricted due to categories having non-independent samples.**

Our analysis procedure allowed us to identify two cutoff points that may be used for targeting of the MDA if one of the subgroups was found to be a significant effect modifier. We did not want to compare different levels of WAZ to each other, but rather those classified as underweight based on a cutoff point to those not classified as underweight. For that reason we compared underweight group to non-underweight group at two different levels.

**Results**
**9. Line 255-257. Using the terminology “the moderate to severe subgroup” and “the severe underweight subgroup”, it currently reads that these are independent subgroups, which they are not. I think some form of rephrasing is needed throughout the paper to avoid giving readers the wrong impression, phrases such as “those either moderate or severely underweight” might help – this removes the words “the” and “subgroup” both of which imply unique membership.**

We are using two different cutoff points of WAZ to compare underweight versus not-underweight, as explained in the response to the comment above. They are not independent subgroups, however the groups we are comparing to each other are independent subgroups. We’ve added more context regarding this in lines 220-225.

**10. Supplementary tables 1-3. Please add the N to these tables.**

Thanks for this suggestion. We’ve added the number of participants in each arm to the tables.

**Discussion**
**11. Results are interpreted accurately, and limitations described. My only suggestion is the same as previous sections – I recommend clarifying the description of the subgroups so that readers do not misinterpret them as being unique. E.g. lines 286-287.**

We’ve added context regarding the non-independence of the subgroups in lines 220-225.

---

## VERSION 2 - REVIEW

---

| | |
|---|---|
| **Reviewer** | **1** |
| **Name** | **von Seidlein, Lorenz** |

**Affiliation** Menzies School of Health Research

**Date** 17-Mar-2025

**COI**

---

The authors have responded adequately to my suggestions.

---

**Reviewer** 3

**Name** Walson, Judd

**Affiliation** University of Washington, Department of Global Health

**Date** 12-Mar-2025

**COI**

---

The authors have adequately addressed the reviewer concerns and comments
